# Trajectories of family functioning and financial toxicity in patients with glioma: a longitudinal study

**DOI:** 10.3389/fpubh.2025.1573000

**Published:** 2025-07-18

**Authors:** Yue Wu, Yihao Wu, Botao Zhou, Yongmou Sun, Rufei Dai

**Affiliations:** ^1^Department of Neurosurgery, The Second Affiliated Hospital of Xuzhou Medical University, Xuzhou, Jiangsu, China; ^2^Department of Neurosurgery, Xuzhou Central Hospital, Xuzhou, Jiangsu, China

**Keywords:** glioma, family functioning, financial toxicity, development trajectory, latent variable growth model, cross-lag model

## Abstract

**Objective:**

This study aims to investigate the longitudinal trajectories of family functioning and financial toxicity in glioma patients, while examining their predictive interrelationships, to establish evidence-based strategies for alleviating economic burden in neuro-oncology care.

**Methods:**

This prospective longitudinal cohort study enrolled 266 glioma patients from the Second Affiliated Hospital of Xuzhou Medical University and Xuzhou Central Hospital (January 2022–June 2024). Family functioning and financial toxicity were serially assessed at three timepoints: baseline (T1, initial diagnosis), 3-month follow-up (T2), and 6-month follow-up (T3). Structural equation modeling (SEM) framework incorporated cross-lagged panel analysis and latent growth curve modeling to examine temporal relationships.

**Results:**

A total of 242 valid consecutive questionnaires were collected. The cross-lagged panel analysis demonstrated that the average family functioning level significantly and positively predicted subsequent financial toxicity at follow-up time points (*β* = 0.478, *p* = 0.01; β = 0.463, *p* < 0.001), while financial toxicity exhibited no significant longitudinal predictive effect on family functioning across subsequent assessments. The latent growth curve modeling revealed parallel declining trajectories: family functioning (slope [S] = −0.410, *p* < 0.001) and financial toxicity (slope [S] = −0.102, *p* < 0.001) both decreased significantly from T1 to T3. At baseline, family functioning showed positive correlation with financial toxicity scores (*r* = 0.377, *p* = 0.002). Initial family functioning level demonstrated dual regulatory effects: (1) negative auto-regulation (*β* = −0.352, *p* = 0.007) and (2) inverse prediction of financial toxicity’s developmental trajectory (*β* = −0.516, *p* = 0.002). Crucially, the initial family functioning level exhibited compensatory effects on financial toxicity dynamics, showing negative coupling with its growth rate (*β* = −0.534, *p* < 0.001).

**Conclusion:**

Family functioning and financial toxicity of glioma patients can predict each other. The initial level of family functioning can positively predict the initial level of financial toxicity, the initial level of family functioning can negatively predict the development speed of itself and financial toxicity, and the development speed of family functioning can positively predict the development speed of financial toxicity.

## Introduction

Glioma is the most common primary intracranial malignant tumor of the central nervous system in adults, accounting for 60–70% of intracranial tumors (1). At present, surgery is the main treatment for glioma, and comprehensive measures such as radiotherapy and chemotherapy are generally provided after diagnosis (2). In addition, the application of advanced therapeutic techniques such as electric field therapy, targeted therapy, immunotherapy, and gene therapy provides new hope for prolonging the survival time of patients (3–5). However, with the continuous progress of treatment technology and the extension of survival period, the treatment cost of patients has gradually increased. At the same time, patients with glioma are often accompanied by motor, sensory, cognitive impairment and other adverse symptoms, such as depression, fatigue, insomnia, pain, etc. (6), and patients lose their ability to work and increase the burden of family care, which leads to heavy psychological burden and economic pressure (7).

Zafar and Abernethy (8) first coined the term “financial toxicity” to characterize the dual economic burden in malignant tumor care: objective financial strain and subjective economic distress. Financial toxicity, prevalent in cancer patients (9), often causes treatment delays/non-adherence and consequent psychosocial harm (10). Glioma of the complexity of the structure and function make the most of the patients with glioma rely on combination of surgery and non-operative treatment, some patients will need to be confirmed after intensive care unit and hospital for a long time, financial toxicity due to direct and indirect treatment costs and impaired ability to work during treatment and rehabilitation is particularly evident in families of glioma patients (11, 12). family functioning reflects the mutual support and close communication between family members, emotional dependency and trouble coping, is considered to promote the patients and their caregivers of positive emotion and posttraumatic growth important family variables. According to McMaster model of family functioning, family as an organic whole, each member has mutual influence. A high level of family functioning not only helps patients and caregivers to carry out effective psychological adjustment when facing disease and disaster, but also helps patients and caregivers to carry out effective psychological adjustment when facing disease and disaster. It can also relieve each other’s stress by promoting emotional communication and coordinated coping (13). Wang et al. (12) reported that family functioning had a promoting effect on the positive psychology and quality of life of lung cancer patients and caregivers, but no research involving financial toxicity was reported. Therefore, we hypothesize that dynamic changes in family functioning levels may negatively predict fluctuations in financial toxicity levels among glioma patients.

Cross-lagged model is a type of structural equation model that examines bidirectional causal relationships between variables and their temporal precedence effects by analyzing lagged effects across different time points. Latent growth model is a type of structural equation model that analyzes repeated measures data through latent growth factors to evaluate individual differences in developmental trajectories over time. The synergy between cross-lagged models and latent growth models lies in their capacity to jointly resolve bidirectional causal relationships and temporal trajectories, effectively addressing measurement errors while disentangling individual heterogeneity, thereby optimizing causal inference validity and model explanatory power.

This study adopted a longitudinal design, and used the cross-lag model and latent growth model to explore the change trajectory and mutual prediction relationship of family functioning and financial toxicity in patients with glioma after diagnosis, so as to provide a theoretical basis for clinical staff to reduce the level of financial toxicity in patients with glioma.

## Subjects and methods

### Subjects

A total of 266 glioma patients admitted to the Second Affiliated Hospital of Xuzhou Medical University and Xuzhou Central Hospital from January 2022 to June 2024 were selected by convenient sampling as the study objects: (1) patients met the clinical diagnostic criteria for glioma and were confirmed by imaging and/or pathology in our hospital; (2) Patients over 18 years old, married, and their spouse over 18 years old; (3) with basic understanding and communication skills, all signed the informed consent form. Exclusion criteria: (1) previous history of mental illness; (2) metastatic glioma, receiving drug or psychological treatment, etc. Exclusion criteria: patients died or withdrew for other reasons.

## Study methods

### Ethics code

This study was conducted after the approval of the Ethics Committee of the Second Affiliated Hospital of Xuzhou Medical University and Xuzhou Central Hospital (approval number: XKZY2021120025).

### Sample size calculation

According to the sample size requirement of the latent growth model, at least 200 cases were required, and the dropout rate of 10% in the longitudinal survey was considered. Therefore, the minimum sample size of this study was set as *n* = 200/ (1–10%) = 223. A total of 266 glioma patients were enrolled in this study.

### Survey tools

1. A self-made questionnaire was used for basic information, including demographic data such as age, gender, marital status, family monthly income, education background, occupation and so on.
2. Family Adaptability and Cohesion Evaluation Scale (FACES). The scale was sinicized and revised by Lipeng et al. (14), including 16 items for cohesion and 14 items for adaptability, with a total of 30 items. Using Liker 5 scale, 1 points means “not,” 5 points means “always,” the total score ranged from 30 to 150, the higher the score, the better the family functioning of the respondents. The Cronbach’s *α* coefficients of the two subscales of family intimacy and adaptability were 0.85 and 0.73, respectively. The Cronbach’s α coefficients of the scale in this study were 0.842.
3. Comprehensive Scores for Financial Toxicity based on the Patient-Reported Outcome Measures; COST-PROM. The scale was developed by de Souza et al. (15) and revised by Yu et al. (16). Positive wealth status (4 items) and negative psychosocial reaction (7 items) were used to investigate the perceived financial toxicity of the respondents. Using the Liker 5 scale, a score of 0 means “not at all” and a score of 4 means “very much.” The total score ranges from 0 to 44, with lower scores indicating more financial toxicity perceived by respondents, and a score of <26 was defined as positive. The Cronbach’s *α* coefficient of the Chinese version of the COST-PROM scale was 0.889, and the test–retest reliability ranged from 0.77 to 0.98. This scale has good reliability and validity and can be used for the study of financial toxicity in the cancer population in China. The Cronbach’s *α* coefficient of the scale in this study was 0.890.

### Data recovery methods

The questionnaire was administered face-to-face in the neurosurgery ward after consent was obtained from the hospital and the patients. The general information questionnaire was obtained at the time of first diagnosis, and the family functioning and financial toxicity questionnaire were obtained at T1 (after the first diagnosis), T2 (3 months after the diagnosis), and T3 (6 months after the diagnosis), respectively. In order to ensure the privacy of patients, the survey was conducted in a confidential environment, and the patients were promised that the questionnaire was only used for this research. The questionnaire was conducted in an anonymous way, and no personal information was disclosed or personal factors were discussed separately. For patients with low education level and dyslexia, the items of the scale were repeated by the investigator, and the patients selected the questionnaire independently.

### Quality control

Three questions with the same question stem and different choice order were placed in different positions of the questionnaire. Invalid questionnaires with three different choices were excluded to ensure the reliability of the questionnaire and to ensure that each questionnaire could express the true will of patients. At the beginning, a total of 266 questionnaires were distributed, and 242 valid consecutive questionnaires were collected after 3 time points, with an effective recovery rate of 90.98%. Missing data were addressed through multiple imputation procedures. Sensitivity analysis showed that the results of lost follow-up data were robust after multiple interpolation (fluctuation of path coefficient < 10%). In order to maintain patients’ enthusiasm for participation, a small gift was given after the questionnaire.

### Statistical methods

Statistical analyses were conducted using SPSS 26.0 and Mplus 8.0. Count data and continuous data were presented as frequencies/percentages and means ± standard deviations, respectively. Pearson correlation analysis was performed to examine inter-variable relationships. Structural equation modeling (SEM) was applied to analyze cross-lagged effects between family functioning and financial toxicity. Unconditional latent linear growth models were used to identify linear developmental trajectories for family functioning and financial toxicity. The intercept represented baseline levels, while the slope denoted temporal changes. Parallel latent growth models were constructed based on univariate models to evaluate time-dependent interactions between family functioning and financial toxicity. Model evaluation was performed using robust maximum likelihood estimation (MLR).

## Results

### General demographic data

A total of 242 patients completed the survey, as shown in Table 1.

**Table 1:** Demographic information of glioma patients (*n* = 242).

Items	Classifications	Number	(%)
Age (years)	< 45	96	39.67
	45 ~ 59	69	28.51
	≥60	77	31.82
Gender	male	115	47.52
	female	127	52.48
Residence	city	94	38.84
	towns	67	27.69
	rural	81	33.47
Monthly family income (yuan)	<4,000	64	26.45
	4,000 ~ 6,000	85	35.12
	6,001 ~ 10,000	66	27.27
	> 10,000	27	11.16
Educational level	primary and below	62	25.62
	junior high school	84	34.71
	senior high school	61	25.21
	colleges and above	35	14.46
Careers	Civil service	10	4.13
	Staff	98	40.50
	Students	7	2.89
	Farmers	60	24.79
	Freelancing	67	27.69

### Common method bias test

The results showed that the variance explained by the first factor in the three measurements was 19.62, 23.16 and 25.38%, respectively, which were less than the critical value of 40%, so there was no obvious common method bias in this study.

### Family functioning and financial toxicity scores of glioma patients at three time points and their correlation analysis

Pearson correlation analysis was used to analyze the family functioning and financial toxicity at three time points, and the results showed that there was a significant correlation between the two at three time nodes (*p* < 0.05), which met the premise of the cross-lag model and the parallel latent variable model. The matrix relationship is shown in Table 2.

**Table 2:** Scores and correlation coefficient matrix of family functioning and financial toxicity in glioma patients at three time points (*r*, *n* = 242).

Items	M	SD	①	②	③	④	⑤	⑥
①family functioning T1	99.57	22.25	1					
② financial toxicity T1	25.92	5.86	0.396^**^	1				
③ family functioning T2	95.69	21.14	0.543^**^	0.226^*^	1			
④financial toxicity T2	25.52	5.92	0.293^**^	0.363^**^	0.464^**^	1		
⑤family functioning T3	88.70	19.03	0.482^**^	0.207^*^	0.523^**^	0.214^**^	1	
⑥financial toxicity T3	24.36	5.47	0.251^**^	0.312^**^	0.332^**^	0.442^**^	0.524^***^	1

^*^p < 0.05, ^**^p < 0.01, ^***^p < 0.001.

### Cross-lagged model of family functioning and financial toxicity in glioma patients

**Figure 1:**
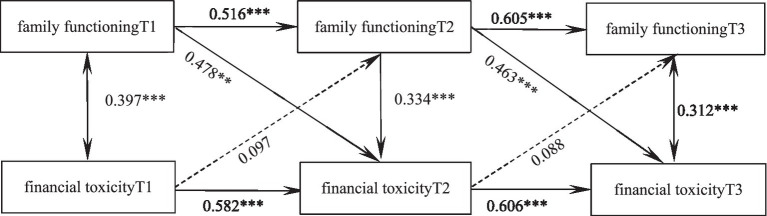
Predictive pathways of family functioning and economic toxicity in 3 time-node glioma patients. *p < 0.05, **p < 0.01, ***p < 0.001.

A cross-lag model was established to investigate the mutual predictive relationship between family functioning and financial toxicity. The model fitted well (*χ^2^/*df = 2.256, GFI = 0.990, TLI = 0.976, RMSEA < 0.001). As shown in Figure 1, the level of family functioning positively predicts the financial toxicity at the next node on average (*β* = 0.478, *p* < 0.01, *β* = 0.463, *p <* 0.001), while the longitudinal prediction effect of financial toxicity on family functioning at the next node is not significant (*β* = 0.097, *p* = 0.137, *β* = 0.088, *p* = 0.151), and the specific path is shown in Figure 1.

### Family functioning and financial toxicity of glioma patients were parallel latent variables

Because the interval time between the three measurements was 3 months, the slope factor loadings of the three measurements were set as 0, 1, and 2, the intercept represents the initial level, and the slope represents the development rate.

### The development trajectory of family functioning in patients with glioma

**Figure 2:**
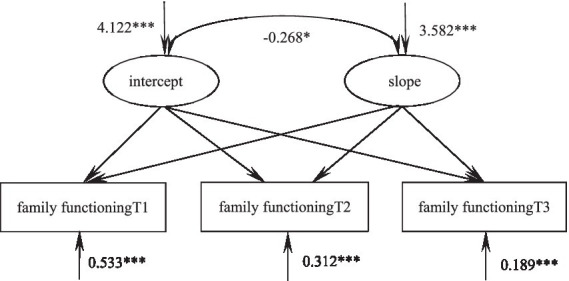
Model of family functioning in glioma patients. *p < 0.05, **p < 0.01, ***p < 0.001.

According to the unconditional latent linear growth model of family functioning of glioma patients, the fitting indicators were as follows: χ*^2^/df* = 2.422, GFI = 0.984, TLI = 0.968, RMSEA = 0.066, SRMR = 0.0353, and the degree of fitting was good. The intercept of the model was 99.57, which was the initial value of family functioning, and the following three measurements showed a downward trend (*S* = −0.410, *p* < 0.001)^.^ There were significant differences in the variation of the intercept (σ^2^ = 4.122, *p* < 0.001) and slope (σ^2^ = 3.582, *p* < 0.001). These results indicated that there were individual differences in the initial level and development speed of family functioning in glioma patients. There was a significant correlation between intercept and slope (*r* = −0.268*, p* = 0.014), suggesting that the higher the initial level of family functioning of glioma patients, the slower the decline rate in the later stage, as shown in Figure 2.

### Trajectories of financial toxicity in glioma patients

**Figure 3:**
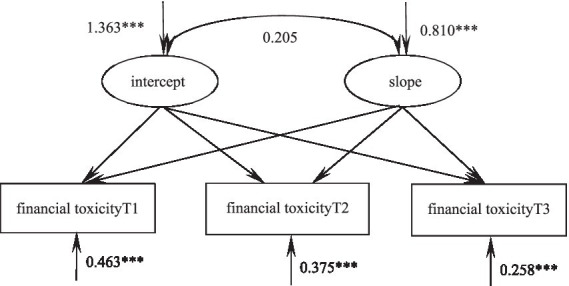
Model of financial toxicity in glioma patients. *p < 0.05, **p < 0.01, ***p < 0.001.

According to the unconditional latent variable linear growth model of financial toxicity in patients with glioma, the fitting indicators were as follows: χ*^2^/df* = 2.440, GFI = 0.978, TLI = 0.982, RMSEA = 0.082, SRMR = 0.020, and the degree of fitting was good. The intercept of the model was the initial value of financial toxicity of 25.92, and the subsequent three measurements showed a downward trend (S = -0.102, *p* < 0.001). There were significant differences in the var*iation* of intercept (σ^2^ = 1.363, *p* < 0.001) and slope (σ^2^ = 0.810, *p* < 0.001). These results indicate that there are individual differences in the initial level and development speed of financial toxicity in patients with glioma. There was no significant correlation between intercept and slope (r = 0.205, *p* = 0.210), suggesting that there was no significant correlation between the initial state of financial toxicity and the development rate of *glioma* patients, as shown in Figure 3.

### Dynamic relationship between family functioning and financial toxicity in glioma patients

**Figure 4:**
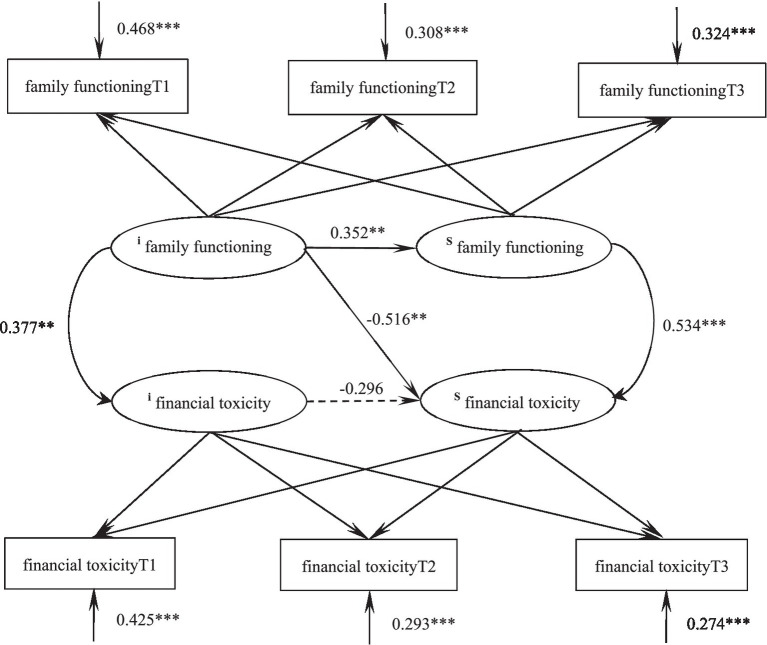
Parallel latent variable model of family functioning and financial toxicity in glioma patients. *p < 0.05, **p < 0.01, ***p < 0.001.

The parallel latent variable growth model of family functioning and financial toxicity of glioma patients was constructed. The fitting indicators were as follows: χ*^2^/df* = 2.415, GFI = 0.978, TLI = 0.966, RMSEA = 0.065, SRMR = 0.037, and the degree of fitting was good. At the initial level, there was a positive correlation between family functioning and financial toxicity score (*r* = 0.377, *p* = 0.002), that is, the higher the level of family functioning of the patient, the higher the financial toxicity score and the lower the financial toxicity level. The initial level of family functioning could negatively predict its development rate (*β* = −0.352, *p* = 0.007), that is, the higher the initial level of family functioning, the slower its decline rate; The initial level of family functioning could negatively predict the development speed of financial toxicity (*β* = −0.516, *p* = 0.002), that is, the higher the initial level of family functioning, the slower the decline of financial toxicity score. The development speed of family functioning could positively predict the development speed of financial toxicity (*β* = 0.534, *p* < 0.001), that is, the faster the decline of family functioning of patients, the slower the decline of financial toxicity score. Figure 4 shows the action path.

## Discussion

Financial toxicity refers to the financial burden and psychological distress of cancer patients during treatment (17), and higher levels of financial toxicity impair patients’ material status, mental health, and quality of life (18). By constructing a cross-lag model, this study found that the level of family functioning positively predicted the financial toxicity at the next node, on average, while the longitudinal predictive effect of financial toxicity on family functioning at the next node was not significant. That is, the higher the level of family functioning, the higher the score of financial toxicity and the lower the level of financial toxicity in the next stage. Harju et al. (19) showed that family functioning status was significantly related to the quality of life of patients. The reason may be that patients with good family functioning, in addition to the necessary life care from the family after illness, their negative emotions, such as anxiety, depression, psychological distress, etc., are effectively output through communication with family caregivers, and then the inner pressure is released. In addition, the higher the level of family functioning of patients after illness, the basic and life care from the family makes the quality of life of patients maintain at the previous level. The stability of the quality of life level is conducive to the better cooperation of patients with treatment, the outcome and prognosis of the disease, the possibility of complications is reduced, and the level of financial toxicity is reduced. Financial toxicity can not provide feedback on the family attributes in the next stage, so financial toxicity can not predict the family functioning in the next stage. This suggests that clinical staff should pay attention to the assessment of family functioning of glioma patients, actively guide and intervene for those with low family functioning level, and provide necessary life care, so as to reduce the reduction of quality of life caused by the disease, which has a positive effect on reducing later financial toxicity.

financial toxicity, which fully reflects the perceived objective financial burden associated with cancer treatment and the resulting subjective psychosocial distress, is another major challenge for patients’ families in addition to the disease (20, 21). family functioning is an important barrier for cancer patients and caregivers to relieve the physical and mental stress caused by the disease. A high level of family functioning can help family members avoid getting into difficulties, promote effective communication, solve problems, and enhance the ability of family members to overcome difficulties. Family functioning is an important indicator of family resources (22). Through the latent growth model, this study found that the scores of family functioning and financial toxicity of glioma patients showed a downward trend in T1-T3 time period, that is, after diagnosis. Zhou et al. (23) showed that good family functioning is a protective factor for suicidal ideation in patients with malignant tumors, indicating that improving the family support system can help prevent suicide and other adverse events in patients with malignant tumors in the future. When a family member suddenly falls ill with a serious disease, affected by the traditional family culture concept, the patient’s family members often respond quickly, using available family resources to do everything possible to help the patient. As a result, the perceived family cohesion of the main caregivers increases rapidly in a short period of time (24). As the treatment continues, the caregivers of patients gradually accept the disease and pay less attention to the patients, which explains the reason for the decline in family functioning. The financial toxicity score of glioma patients showed a downward trend, that is, the level of financial toxicity showed an upward trend. The reason may be due to Chinese traditional culture and current medical practice. Domestic medical staff usually disclose cancer-related information (cancer diagnosis, cancer stage, disease prognosis, etc.) to caregivers of patients rather than patients. Then the family caregivers decide whether to inform the patients and what content should be disclosed to the patients, which leads to a low understanding of the disease in the early stage of the disease. With the progress of the disease treatment, the understanding of glioma gradually increases, and then the level of financial toxicity increases.

The results of the parallel latent growth model showed that at the initial level, family functioning was positively correlated with the financial toxicity score, that is, the higher the level of family functioning of the patient, the higher the financial toxicity score and the lower the financial toxicity level. This is consistent with the results of previous studies (25). The initial level of family functioning can negatively predict the development rate of itself and financial toxicity, that is, patients with a higher initial level of family functioning have a slower decline rate and a slower decline rate of financial toxicity scores. When patients face the high cost of cancer treatment, the characteristics of family functioning such as cohesion and adaptability can help encourage patients to rely on family as their spiritual support, and obtain the support of the overall strength of the family to produce positive emotions and adopt positive coping styles. Reducing the economic and psychological burden (26); Patients with poor family functioning do not feel sufficient love and care from other family members, and are more likely to have negative emotions when facing the loss of work ability and the financial difficulties brought by the disease treatment. and further aggravate the subjective financial toxicity (27). The development speed of family functioning can positively predict the development speed of financial toxicity, that is, the faster the decline of family functioning of patients, the slower the decline of financial toxicity score. Caregivers with good family functioning can establish a warm mutuality with patients, which in turn enables patients to feel the sense of kinship care and disease control, generate more positive emotional feelings, alleviate patients’ negative emotions, and improve their negative expectations of treatment costs (28). The faster the decline in family functioning, the less sense of security patients can get, the less effective output of negative emotions, and the faster the decline in financial toxicity scores.

## Deficiencies and prospects

This longitudinal study investigated the trajectories of family functioning and financial toxicity among 242 glioma patients within 6 months post-diagnosis, providing a basis for clinically dynamic monitoring of these constructs. However, this study has several limitations: First, the use of convenience sampling may limit the generalizability of findings; second, cultural factors specific to China might restrict the applicability of results internationally; third, the relatively short follow-up period (6 months) and prolonged research cycle, combined with a limited sample size, may reduce statistical power. Additionally, financial toxicity in glioma patients is influenced by multiple factors (e.g., pre-existing mental health conditions, social support networks, tumour grade), which were not fully controlled in this study. Future work will address these limitations through multi-center large-sample studies with extended longitudinal follow-up periods, while implementing rigorous control of confounding factors to better elucidate the relationship between family functioning and financial toxicity.

## Conclusion

The family functioning and financial toxicity of glioma patients have a reciprocal predictive effect, indicating that the family functioning of glioma patients is closely related to the financial toxicity. This bidirectional predictive relationship indicates that early enhancement of family functioning may decelerate financial toxicity progression. Clinicians should prioritize dynamic monitoring of both constructs, as improving baseline family functioning could simultaneously mitigate initial economic strain and slow their mutual deterioration, thereby optimizing long-term patient outcomes.

## Data Availability

The original contributions presented in the study are included in the article/supplementary material, further inquiries can be directed to the corresponding author.
